# Major Phenolic Compounds, Antioxidant Capacity and Antidiabetic Potential of Rice Bean (*Vigna umbellata* L.) in China

**DOI:** 10.3390/ijms13032707

**Published:** 2012-02-29

**Authors:** Yang Yao, Xu-Zhen Cheng, Li-Xia Wang, Su-Hua Wang, Guixing Ren

**Affiliations:** Institute of Crop Science, Chinese Academy of Agricultural Sciences, South Xueyuan Road, Haidian District No.80, Beijing 100081, China; E-Mails: yaoyang@caas.net.cn (Y.Y.); chengxz@caas.net.cn (X.-Z.C.); wanglx@caas.net.cn (L.-X.W); wangsh@caas.net.cn (S.-H.W.)

**Keywords:** rice bean, antioxidant, α-glucosidase inhibitory, advanced glycation end products

## Abstract

Interest in edible beans as nutraceuticals is increasing. In the present study, the individual phenolic acids, the total phenolic content (TPC), the total flavonoid content (TFC), and the antioxidant and antidiabetic potential of 13 varieties of rice beans from China were investigated. Eight phenolic compounds (catechin, epicatechin, *p*-coumaric acid, ferulic acid, vitexin, isovitexin, sinapic acid, quercetin) were analyzed on an ultra-performance liquid chromatography (UPLC) mass spectrometry (MS) system. The rice bean varieties had significant differences in total phenolic compounds (ranging from 123.09 ± 10.35 to 843.75 ± 30.15 μg/g), in TPC (ranging from 3.27 ± 0.04 to 6.43 ± 0.25 mg gallic acid equivalents (GAE)/g), in TFC (ranging from 55.95 ± 11.16 to 320.39 ± 31.77 mg catechin (CE)/g), in antioxidant activity (ranging from 39.87 ± 1.37 to 46.40 ± 2.18 μM·TE/g), in α-glucosidase inhibition activity (ranging from 44.32 ± 2.12 to 68.71 ± 2.19) and in advanced glycation end products formation inhibition activity (ranging from 34.11 ± 0.59 to 75.75 ± 0.33). This study is the first report on phytochemistry and biological activities in rice beans.

## 1. Introduction

Antioxidants refer to compounds possessing free radical-scavenging activity, transition metal-chelating activity, and/or singlet oxygen-quenching capacity [1,2]. Several studies have suggested that the cells of diabetic patients are under oxidative stress with an imbalance between free radical-generating and radical-scavenging capacities. The increased free radical production and reduced antioxidant defence may partially mediate the initiation and progression of diabetes-associated complications [3,4].

Acting as a key enzyme for carbohydrate digestion, intestinal α-glucosidase is one of the glucosidases located at the epithelium of the small intestine. Alpha-Glucosidase has been recognised as a therapeutic target for modulation of postprandial hyperglycaemia, which is the earliest metabolic abnormality to occur in type II diabetes mellitus [5,6]. Inhibition of intestinal α-glucosidases delays the digestion and absorption of carbohydrates, thereby suppressing postprandial hyperglycaemia [7]. Hyperglycemia that occurs in diabetic conditions favors glucose metabolism through the polyol pathway and promotes advanced glycation end-products (AGE) formation, which is implicated in the pathogenesis of diabetic nephropathy and other complications. Thus, the discovery and investigation of AGE inhibitors offers a potential therapeutic approach for the prevention of diabetic complications [8,9].

Rice bean (*Vigna umbellata* L.), known as climbing mountain bean, mambi bean and oriental bean, is native to Southeast Asia [10,11]. Our previous study found that rice bean exhibited excellent antioxidant capacity and antidiabetic potential out of sixteen species of beans [12]. Despite several studies on nutritional factors (protein, fat, starch and calcium) in some rice beans [13], a systematic comparison of different varieties on their phytochemistry and biological activities is lacking. Therefore, the present study was carried out with the following aims: (1) to identify and quantify the individual phenolic compounds and measures the total phenolic content (TPC) and total flavonoid content (TFC) in thirteen rice bean varieties; (2) to assess the relative antioxidant, α-glucosidase inhibitory and advanced glycation end products formation inhibitory activities of rice beans; and (3) to investigate the correlations between phytochemicals and biological activities. This study is the first report on the application of an ultra performance liquid chromatography (UPLC) for the rapid separation and quantification of individual phenolic acids in rice bean.

## 2. Materials and Methods

### 2.1. Materials

Thirteen varieties of rice beans were provided by the Chinese National Genebank (Beijing, China). Trolox, catechin, epicatechin, *p*-coumaric acid, ferulic acid, vitexin, isovitexin, sinapic acid, quercetin, 1,1-diphenyl-2-picrylhydrazyl (DPPH) radical, Folin-Ciocalteu reagent, rat intestinal acetone powder, bovine serum albumin (BSA), D-glucose and methylglyoxal (MGO) were all purchased from Sigma-Aldrich (St. Louis, MO, USA). All of the chemicals were of analytical grade and were obtained from Beijing Chemical Reagent (Beijing,China). All of the analytical grade solvents for high performance liquid chromatography (HPLC) were purchased from Fisher Chemicals (Shanghai, China).

### 2.2. Extraction

All dried samples were ground in a laboratory mill and passed through a sieve (80 mesh). Bean samples (10 g) were extracted twice in 100 mL of 70% ethanol for 2 h at room temperature. After vacuum filtration, the supernatants were combined and concentrated under reduced pressure in a rotary evaporator at 50 °C. After freeze-drying, the sample powder was stored at −20 °C until analysis. The biological activities of the rice bean were measured at a concentration of 10 mg/mL.

### 2.3. Determination of Total Phenolic Content (TPC)

TPC was measured using the Folin-Ciocalteu method as previously described by Zhou, Fang, Lü, Chen, Liu, and Ye [14] and modified by Yao and Ren [15]. Briefly, 50 μL of the extract was mixed in 5 mL of distilled deionised water followed by the addition of 500 μL of 1 M Folin-Ciocalteu reagent and 500 μL of a 20% (w/v) Na_2_CO_3_ solution. The mixture was thoroughly mixed and allowed to stand for 60 min at room temperature before reading the absorbance at 765 nm (Bio-Rad Smart Spec Plus Spectrophotometer, Hercules, USA). Quantification was performed with respect to the standard curve of gallic acid. The results were expressed as milligrams of gallic acid equivalent (GAE) per gram.

### 2.4. Determination of Total Flavonoid Content (TFC)

TFC was measured using the colorimetric method as previously described by Zou, Lu, and Wei [16] with some modifications. The extract (0.5 mL) was mixed in 2 mL of distilled water followed by the addition of 0.15 mL of a 5% (w/v) NaNO_2_ solution. After 6 min, 0.15 mL of a 10% AlCl_3_ solution was added to the mixture and the solution was then allowed to stand for 6 min followed by the addition of 2 mL of a 4% NaOH solution. Water was immediately added to bring the final volume to 5 mL, and the mixture was then thoroughly mixed and allowed to stand for an additional 15 min before the absorbance was measured. The results were expressed as micrograms of catechin equivalent (CE) per gram.

### 2.5. UPLC-MS Analysis of Individual Phenolic Acids

The chromatographic system consisted of an ultra performance liquid chromatography system equipped with a Triple Quadrupole Mass Spectrometer (TQD) (Waters Milliford, MA, USA). The analytical column was a Waters ACQUITY UPLC BEH Shield RP18 (2.1 mm × 100 mm; 1.7 μm) column. The following mobile phase consisting of methanol (A) and 0.1% (v/v) formic acid in water (B) was used in a gradient elution: 0–5 min with 35–40% solution A; 5–8 min with 40–65% solution A; 8–10 min with 65–90% solution A; 10–11 min with 90% solution A; 11–13 min with 90–35% solution A; and 13–15 min with 35% solution A. The flow rate was 0.25 mL/min, and the injection volume was 10 μL. The column and autosampler were maintained at 35 °C and 10 °C, respectively. Identification and quantification of phenolic compounds were performed by comparing the retention times of authentic standards and the areas of external standards.

### 2.6. Evaluation of Total Antioxidant Activity Using the DPPH (1,1-Diphenyl-2-picryl hydrazyl) Method

The DPPH radical-scavenging activity was determined using the method reported by Yao, Sang, Zhou, and Ren [17]. DPPH (100 μM) was dissolved in 96% ethanol. The DPPH solution (1 mL) was mixed with 1 mL of the extract solution. The mixture was shaken and allowed to stand at room temperature in the dark for 10 min. The decrease in absorbance of the resulting solution was measured at 517 nm after 10 min. The results were expressed in micromoles of Trolox equivalents (TE) per gram.

### 2.7. Measurement of Alpha-Glucosidase Inhibition Activity

The α-glucosidase inhibition activity was determined as previously described with slight modifications [18]. Alpha -Glucosidase (1 U/mL) inhibition activity was assayed using 50 μL of extracts with varying concentrations incubated with 100 μL of 0.1 M phosphate buffer (pH 7.0) in 96-well plates at 37 °C for 10 min. After preincubation, 50 μL of 5 mM p-nitrophenyl-α-D-glucopyranoside in a 0.1 M phosphate buffer (pH 7.0) was added to each well. The reaction mixtures were incubated at 37 °C for 5 min. The absorbance readings were recorded at 490 nm on a microplate reader before and after incubation (BioRad, IMAX, Hercules, CA, USA). The results were expressed as a percent of α-glucosidase inhibition, and the inhibition activity was calculated according to the following equation: (*A*_control_ − *A*_sample_)/*A*_control_ × 100%. The IC_50_ value was defined as the concentration of bean extracts required to inhibit 50% of the enzyme activity.

### 2.8. Evaluation of Glycation End Product (AGE) Inhibition Activity

BSA-glucose and BSA-MGO models were used for the evaluation of the inhibition effect of the extracts on the formation of advanced glycation end products. The BSA-glucose assay was carried out according to the method reported by Peng *et al.* [19]. Briefly, 5 g of BSA and 14.4 g of D-glucose were dissolved in 1.5 M phosphate buffer (pH 7.4) to obtain the control solution with 50 mg/mL BSA and 0.8 M D-glucose. Two millilitres of the control solution were incubated at 37 °C for 7 days in the presence or absence of 1 mL of bean extracts in a 1.5 M phosphate buffer (pH 7.4). After 7 days of incubation, the fluorescent intensity (excitation at 330 nm and emission at 410 nm) was measured. Percent inhibition of AGE formation by each extract was calculated using the following equation: (1 − (fluorescence of the solution with inhibitors/fluorescence of the solution without inhibitors)) × 100%.

The BSA-MGO assay was carried out: 40 mg of BSA was mixed with 31 μL of MGO in a 0.1 M phosphate buffer (pH 7.4) to obtain the control solution with 1 mg/mL BSA and 5 mM MGO. Two millilitres of the control solution were incubated at 37 °C for 6 days with or without 1 mL of the bean extracts in phosphate buffer. The percent inhibition was calculated based on the equation applied in the BSA-glucose assay as described above (excitation at 340 nm and emission at 420 nm).

### 2.9. Statistical Analysis

All values were expressed as mean ± SD. Data were analysed using one-way analysis of variance (ANOVA) followed by the Tukey-Kramer test (Matlab version 7.6).

## 3. Results and Discussion

### 3.1. Phytochemical Content

Typical phenolics that possess antioxidant activity are known to be mainly phenolic acids and flavonoids. Phenolic acids and flavonoids are major classes of phenolic compounds that widely occur in the plant kingdom [14]. Beans are normally cooked in water and therefore water extraction is an appropriate method for identification of specific and relevant phenolic compounds [20]. Eight standards were separately detected within 15 min. Three phenolic acids (*p*-coumaric acid, ferulic acid, and sinapic acid) and five flavonoids (catechin, epicatechin, vitexin, isovitexin and quercetin) were determined in the different rice bean samples (Table 1). Vitexin was the dominant phenolic compound in all varieties, followed by catechin and isovitexin. The highest vitexin and isovitexin contents were both found in the D0000090 variety (401.84 ± 21.57 μg/g and 190.29 ± 3.69 μg/g, respectively). For catechin levels, the highest was found in the D0000958 variety, which was 7.4 times higher than the lowest level found in the D0000809 variety. Epicatechin, *p*-coumaric acid, ferulic acid, sinapic acid and quercetin concentrations were similar to each other. The total phenolic compounds (TP) reported in Table 1 are the sum of catechin, epicatechin, *p*-coumaric acid, ferulic acid, vitexin, isovitexin, sinapic acid and quercetin. Among all of the studied rice beans, the D0000090 (843.75 ± 30.15 μg/g) variety possessed the highest TP, which was 6.9 times greater than that of the D0000318 variety (123.09 ± 10.35 μg/g). Rice bean (*Vigna umbellate*) and adzuki bean (*Vigna angularis*) are phylogenetically closely related, and have low level of divergence based on nucleotide sequences, seed proteins and isozymes [21,22]. Due to absence of reported studies on the phytochemicals and biological activities of the rice bean, we have had to compare some of the data with those reported in adzuki beans.

The TPC, as measured by the Folin-Ciocalteu method, varied among different rice beans (Table 2). In the present study, the D0000090 variety with an average TPC of 6.43 ± 0.25 mg GAE/g was found to possess the highest TPC among all of the studied varieties and this variety had a TPC value that was 2.0 times greater than that of the D000318 variety (3.27 ± 0.04 mg GAE/g). With regard to TFC levels, the highest TFC level was also found in the D0000090 variety, which was two times higher than the lowest level found in the D0000318 variety. Previous studies [23–25] have reported that the differences between various cultivars are likely due to genotypic and environmental differences including location, UV-B irradiation, diseases and pest exposure, choices of parts tested, and times of taking samples.

### 3.2. Antioxidant Activity

The antioxidant activities of rice bean extracts were evaluated by measuring their DPPH radical-scavenging activities. All of the extracts exhibited antioxidant activities (Table 3). The D0000874 variety had the greatest DPPH free radical- scavenging capacity, and the D0000318 variety had the least DPPH free radical- scavenging capacity. All rice bean varieties had antioxidant values between 39.87 and 46.40 μM TE/g. The relatively stable organic radical, DPPH, has been widely used in the determination of antioxidant activity of single compounds and different plant extracts. Although the DPPH method is simple and rapid, it generally has a relatively small linear reaction range [26]. Chou, Chao, and Chuang [27] reported that at a dosage of 0.62 to 10 mg/mL, the adzuki bean extracts showed 22.2 to 80.2% scavenging activity of DPPH and the activity increased with the concentration of the extracts. Phenolic compounds are considered to be the major compounds contributing to the total antioxidant activities of the beans [28]. In this study, the TPC and TFC were highly correlated with their antioxidant capacity (*p* < 0.05) (Table 4). A similar effect has been observed by Yao, Sang, Zhou, and Ren [18], who investigated seven colored grains and found that the antioxidant activity in the grains was positively correlated with TPC. Rice bean and adzuki bean have a similar color of seed coats. Lin and Lai [20] have reported that adzuki bean seed coats mainly contained proanthocyanins, which are a group of polyphenolic bioflavonoids that contribute to the high antioxidant abilities.

### 3.3. Alpha-Glucosidase Inhibition Activities

To determine the α-glucosidase inhibition ability of legums *in vitro*, we measured the ability of the extracts to inhibit α-glucosidase at a concentration of 10 mg/mL (Table 3). The D0000310 variety was the most active (68.71%) followed by the D0000294 variety (66.06%). Itoh *et al.* [29] investigated the antidiabetic effects of adzuki bean on streptozotocin (STZ)-induced diabetic rats, and suggested that the active fraction of adzuki bean suppresses the postprandial blood glucose by inhibiting α-glucosidase. Alpha-Glucosidase inhibition was not correlated with the phenolic acids in the extracts (Table 4). Red and pink pigments in the seed coats of beans, known as anthocyanins, can directly induce the secretion of insulin from pancreatic cells in *ex vivo* assays [14,30]. Mccue, Kwon, and Shetty [31] investigated fifteen Asian beans, fruits and vegetables, and they concluded that a high phenolic content does not always confer a high inhibition of α-glucosidase activity of a food extract, which may in act be due to the nonphenolic compounds in the samples.

### 3.4. Advanced Glycation End products Formation Inhibition Activities

BSA-glucose and BSA-MGO models were used for the evaluation of the inhibitory effect on the formation of advanced glycation end products. The results are shown in Table 3. In the present study, the inhibition ability of the D0001152 variety was higher than 65% in both of the BSA-glucose and BSA-MGO assays. The results (Table 4) obtained in the present study showed that the results from the BSA-MGO and BSA-glucose assays are significantly correlated with TPC and TFC assays (*p* < 0.05). Similar results have been observed by Peng *et al.* [19] who investigated the correlation of the total phenolic content and the inhibitory effect of the phenolics on the formation of advanced glycation end products of mung bean, black bean, soybean and cowpea. They demonstrated that phenolic compounds inhibit the formation of advanced glycation end products by inhibition of free radical generation in the glycation process and subsequent inhibition of protein modification.

## 4. Conclusion

In conclusion, rice beans have been shown to possess significant differences in phytochemical content, antioxidant activities, α-glucosidase inhibition activities and advanced glycation end product formation inhibitory activities.

## Figures and Tables

**Table 1:** Content of individual phenolic compounds in rice beans (in μg/g).

	*p*-Coumaric acid	Ferulic acid	Sinapic acid	Catechin	Epicatechin	Vitexin	Isovitexin	Quercetin	Total
D0000318	5.67 ± 0.49 i	11.57 ± 0.96 j	nd	53.48 ± 3.29 f,g	nd	26.46 ± 1.92 e,f	7.45 ± 0.68 g	18.46 ± 1.25 d	123.09 ± 10.35 f
D0000708	17.84 ± 1.28 e,f,g	13.20 ± 1.32 j	17.21 ± 1.24 d	142.19 ± 8.96 b	4.37 ± 0.48 d	30.68 ± 1.84 e	nd	27.15 ± 2.13 b,c	252.64 ± 13.62 e
D0000294	16.58 ± 1.48 f,g	28.15 ± 1.45 f,g	19.68 ± 1.53 c,d	78.51 ± 5.47 d,e	6.89 ± 0.96 c	nd	0.43 ± 0.03 h	10.77 ± 0.98 e	150.24 ± 17.28 f
D0000874	31.25 ± 2.41 b	49.71 ± 2.48 c	21.35 ± 1.64 c	132.68 ± 11.20 b	11.24 ± 1.04 a	33.13 ± 1.64 e	27.08 ± 1.59 e,f	26.53 ± 1.63 b,c	332.97 ± 14.54 d
D0000955	21.74 ± 1.92 d,e	25.30 ± 1.93 g,h	17.33 ± 1.38 d	69.33 ± 5.42 e,f	1.35 ± 0.24 e	30.64 ± 1.88 e	118.04 ± 15.75 d	35.46 ± 2.35 a	219.19 ± 21.32 e
D0000958	25.32 ± 1.47 c,d	31.48 ± 2.10 e,f	18.29 ± 2.53 c,d	182.64 ± 12.03 a	5.49 ± 0.79 d	40.16 ± 1.29 e	nd	29.31 ± 2.47 b	332.69 ± 25.67 d
D0000699	20.09 ± 2.21 e,f	54.63 ± 1.48 b	25.32 ± 1.29 b	89.72 ± 4.12 c,d	7.13 ± 0.65 c	192.53 ± 16.68 c	105.34 ± 8.74 d	12.35 ± 1.08 e	507.11 ± 19.44 c
D0000090	39.72 ± 2.69 a	78.32 ± 3.59 a	27.35 ± 1.97 b	68.53 ± 3.95 e,f	2.29 ± 0.11 e	401.84 ± 21.57 a	190.29 ± 3.69 b	35.41 ± 2.84 a	843.75 ± 30.15 a
D0001152	28.15 ± 2.45 b,c	19.65 ± 1.29 i	8.42 ± 0.86 e	43.59 ± 6.87 g	nd	250.71 ± 17.62 b	271.97 ± 9.98 a	24.63 ± 2.36 c	647.12 ± 16.32 b
D0000310	21.36 ± 1.40 d,e	21.08 ± 1.46 h,i	31.08 ± 1.98 a	175.39 ± 14.2 a,1	9.77 ± 0.72 b	186.39 ± 18.19 c	33.27 ± 1.08 e	17.1 ± 1.28 d	495.44 ± 19.50 c
D0000329	19.73 ± 2.10 e,f	41.15 ± 2.21 d	11.27 ± 1.27 e	54.28 ± 8.60 f,g	4.68 ± 0.38 d	nd	23.99 ± 1.31 e,f	nd	155.10 ± 14.29 f
D0000809	15.40 ± 1.07 g,h	48.07 ± 2.08 c	8.16 ± 0.75 e	24.76 ± 1.29 h	7.10 ± 0.56 c	73.81 ± 2.81 d	18.85 ± 0.46 f,g	30.54 ± 2.69 b	226.69 ± 28.17 e
D0000651	11.29 ± 1.14 h	35.86 ± 2.74 e	8.41 ± 0.86 e	101.17 ± 8.39 c	nd	232.92 ± 17.16 b	143.11 ± 5.51 c	nd	232.76 ± 15.46 e

Data are expressed as mean ± standard deviation of triplicate samples. Means in a column with different letters differ significantly (*p* < 0.01).

**Table 2:** Total phenolic contents (TPC) and total flavonoid content (TFC) of rice beans.

	TPC	TFC
D0000318	3.27 ± 0.04 h	55.95 ± 11.16 h
D0000708	5.02 ± 0.14 c,d	211.76 ± 25.53 c,d
D0000294	5.14 ± 0.09 c,d	209.75 ± 22.79 c,d
D0000874	6.02 ± 0.25 a,b	294.52 ± 22.05 a,b
D0000955	3.92 ± 0.13 g,f,h	63.99 ± 37.44 g,h
D0000958	5.52 ± 0.24 d,e,f	167.69 ± 12.89 d,e
D0000699	5.44 ± 0.15 b,c	254.83 ± 23.96 b,c
D0000090	6.43 ± 0.25 a	320.39 ± 31.77 a
D0001152	4.28 ± 0.10 e,f,g	146.20 ± 23.76 e,f
D0000310	4.95 ± 0.15 c,d,e	218.80 ± 12.95 c,d
D0000329	4.11 ± 0.14 e,f,g	140.35 ± 13.78 e,f
D0000809	3.75 ± 0.04 g,h	115.84 ± 13.32 e,f,g
D0000651	3.89 ± 0.14 g,f,h	112.00 ± 29.97 f,g

Data are expressed as mean ± standard deviation of triplicate samples. TPC was expressed as mg GAE/g. TFC was expressed as μg CE/g. Means in a column with different letters differ significantly (*p* < 0.01).

**Table 3:** Biological activities of rice beans.

	DPPH	α-Glucosidase inhibition(%)	BSA-GLUCOSE	BSA-MGO
D0000318	39.87 ± 1.37 e	52.49 ± 1.88 e,f	36.15 ± 0.52 a	34.11 ± 0.59 j
D0000708	45.76 ± 1.42 a,b	61.82 ± 2.68 b,c	61.42 ± 0.04 f	58.82 ± 1.48 c,d
D0000294	41.08 ± 2.11 c,d,e	66.06 ± 2.79 a,b	60.86 ± 0.70 f	48.28 ± 1.72 f
D0000874	46.40 ± 2.18 a	60.01 ± 3.51 c,d	71.63 ± 0.73 b	55.32 ± 1.27 e
D0000955	40.87 ± 0.99 c,d,e	55.45 ± 2.46 d,e	57.27 ± 0.68 g	45.96 ± 1.16 f,g
D0000958	43.71 ± 1.40 a,b,c,d	49.11 ± 2.65 f,g	55.02 ± 0.34 h	42.10 ± 1.67 I,h
D0000699	44.55 ± 1.34 a,b,c	47.16 ± 2.49 f,g	69.20 ± 0.28 c	59.70 ± 1.77 c
D0000090	46.20 ± 1.68 a	46.69 ± 3.71 f,g	70.34 ± 0.51 c	72.06 ± 0.85 a
D0001152	45.24 ± 2.09 a,b	60.06 ± 2.70 c,d	75.75 ± 0.33 a	66.23 ± 0.87 b
D0000310	46.29 ± 2.03 a	68.71 ± 2.19 a	65.54 ± 0.47 d	57.25 ± 0.45 c,d,e
D0000329	42.87 ± 1.61 a,b,c,d,e	48.68 ± 2.15 f,g	61.91 ± 0.44 f	43.65 ± 0.61 g,h
D0000809	42.13 ± 1.45 b,c,d,e	45.23 ± 2.28 g	53.34 ± 0.19 i	56.98 ± 0.69 d,e
D0000651	40.14 ± 1.58 d,e	44.32 ± 2.12 g	63.51 ± 0.84 e	40.47 ± 0.41 i

Data are expressed as mean ± standard deviation of triplicate samples. The anti-DPPH capacity was expressed as μM TE/g. Means in a column with different letters differ significantly (*p* < 0.01). Bovine serum albumin (BSA), D-glucose and methylglyoxal (MGO), 1,1-diphenyl-2-picryl hydrazyl (DPPH).

**Table 4:** Correlation coefficient of total phenolics acid, DPPH, α-glucosidase inhibition, BSA-MGO, BSA-Glucose and tyrosinase inhibition assay.

	TPC	TFC	DPPH	α-Glucosidase inhition	BSA-Glucose	BSA-MGO
TP	0.612 a	0.617 a	0.707 b	−0.024	0.663 a	0.825 b
TPC		0.980 b	0.750 b	0.216	0.668 a	0.670 a
TFC			0.792 b	0.200	0.667 a	0.684 b
DPPH				0.323	0.697	0.778 b
α-Glucosidase inhibition					0.193	0.156
BSA-Glucose						0.728 b

a Correlation is significant at *p* < 0.05 level (2-tailed).

b Correlation is significant at *p* < 0.01 level (2-tailed).
